# Sex hormone binding globulin (SHBG) modulates mitochondrial dynamics in PPARγ-depleted equine adipose derived stromal cells

**DOI:** 10.1007/s00109-024-02459-z

**Published:** 2024-06-14

**Authors:** Krzysztof Marycz, Benita Wiatrak, Jennifer M. Irwin-Houston, Klaudia Marcinkowska, Malwina Mularczyk, Lynda Bourebaba

**Affiliations:** 1 Department of Experimental Biology, Faculty of Biology and Animal Science, Wrocław University of Environmental and Life Sciences, Norwida 27B, 50-375 Wrocław, Poland; 2 International Institute of Translational Medicine, Jesionowa 11, Malin, 55-114 Wisznia Mała, Poland; 3 Department of Medicine and Epidemiology, School of Veterinary Medicine, University of California, Davis, Davis, CA 95516 USA

**Keywords:** PPARγ, SHBG, Equine ASCs, Metabolic syndrome, Mitochondria, Senescence

## Abstract

**Abstract:**

Peroxisome proliferator-activated receptor gamma (PPARγ) is a transcription factor that promotes adipogenesis, lipid uptake and storage, insulin sensitivity, and glucose metabolism. Hence, defects in PPARγ have been associated to the development of metabolic disorders. Sex hormone-binding globulin (SHBG) is a glycoprotein primarily produced in the liver that regulates the bioavailability of sex hormones. Alike PPARγ, low SHBG levels have been correlated with insulin resistance and associated endocrine abnormalities. Therefore, this study aimed to verify whether SHBG may restore depleted PPARγ functions and thus serve as a new candidate for the management of metabolic conditions. A model of equine adipose-derived stromal cells (EqASCs) has been used, in which a PPARγ silencing and SHBG treatment have been achieved to determine the changes in cell viability, premature senescence, oxidative stress, and mitochondrial functions. Obtained data demonstrated that loss in PPARγ triggers cell apoptosis which is not reversed by SHBG application. Moreover, PPARγ knockdown cells exhibited premature senescence, which has been substantially alleviated by SHBG concomitantly to increased BAX/BCL2 ratio, suggesting a possible effect on senescence-induced apoptosis resistance. Interestingly, PPARγ silencing induced a significant alteration in mitochondrial membrane potential as well as the expression of dynamics and metabolism-related markers. SHBG treatment enabled to ameliorate the transmembrane potential, to normalize the expression levels of key dynamics and metabolism mediators, and to restore the protein levels of PINK, which is critically involved in mitochondria recycling machinery. Presented data suggest that SHBG may provide new mechanistic insights into the regulation of PPARγ functions, and thus offers a preliminary picture on a possible SHBG-PPARγ metabolic crosstalk.

****Key messages**:**

- PPARγ is a transcription factor that tightly regulates cell metabolism.
- Low SHBG levels correlate with insulin resistance and associated endocrine abnormalities.
- PPARγ silencing reduces cell viability, triggers premature senescence and profound mitochondrial failure in equine ASCs.
- SHBG protein reverses senescent phenotype and apoptosis resistance of PPARγ- ASCs.
- SHBG improves mitochondrial dynamics and metabolism following PPARγ knockdown.
- SHBG might serve as a PPARγ potential mimicking agent for the modulation of ASCs metabolic processes.

## Introduction

Metabolic flexibility is significantly impacted by glucose metabolism and sensitivity to insulin, that are two fundamental factors maintaining the body metabolism homeostasis. Excessive caloric intake, accumulation of reactive lipids combined with limited physical activity have been shown to be strongly involved in aging, insulin resistance, type 2 diabetes, and cardiometabolic diseases, all of which being directly associated with obesity or adipose tissue dysfunction, and systemic glucose metabolism deterioration [1]. These endocrine abnormalities if not treated, culminate to metabolic syndrome development, in which the key pathophysiological components include abdominal fat accumulation, reduced systemic insulin sensitivity, low-grade inflammation, high blood pressure, high blood sugar levels, high triglyceride levels, and low levels of high-density lipoprotein (HDL) cholesterol [2]. Thus, adipose tissue metabolic and hormonal homeostasis, as well as adipocyte differentiation potential and secretory activity are critically important for identifying molecular therapeutic targets that might mediate obesity and metabolic-associated diseases [3]. 

Adipose tissue is no longer recognized only as a passive energy storage organ but is a fully functional endocrine regulator that modulates systemic glucose uptake and insulin sensitivity by releasing a number of adipokines, cytokines, hormones, and other molecules that impact endocrine homeostasis [4]. For that reason, adipose tissue plasticity is critically associated with metabolic oscillation, however still little is known about important components linking metabolic homeostasis of adipose tissue and metabolic inflexibility. Peroxisome proliferator-activated receptor gamma (PPARγ) is recognized as one of the key metabolic regulators of adipose tissue metabolic homeostasis modulating lipid synthesis, hormonal response, glucose metabolism, and adipokines production among many other functions [5]. PPARγ is a member of the nuclear receptor (NR) superfamily of ligand-activated transcription factors (TFs) involved in the regulation of essential metabolic pathways associated with adipocyte formation, lipogenesis, adipocyte function, and survival [6]. PPARγ is highly expressed in adipose tissue and adipose stromal cells where it plays an important role in the modulation of adipose tissue inflammation, and hormonal homeostasis. Loss of PPARγ function has been shown to promote insulin resistance, suggesting a critical role for PPARγ in insulin signalling associated with adipose tissue metabolism [7]. These findings have also been supported by animal studies, where PPARγ-null mice exhibited a higher sensitivity for insulin than wild-type mice, and what is more depletion of PPARγ protected against high-fat diet-induced insulin resistance However, still little is known regarding the modulation of the post-transcriptional activity of PPARγ that might modulate adipose tissue metabolic and hormonal function [8–10].

PPARγ as a molecular regulator of fatty acid oxidation in adipose tissue plays also an important role in controlling oxidative metabolism, glucose utilization, and mitochondrial OXPHOS [11]. A growing body of evidence suggests that PPARγ may translocate to mitochondria and modulate cell- or tissue-specific function including adipocyte differentiation, expansion, and survival [12]. Adipocyte mitochondria use fat for energy production and deterioration of its function has been implicated in abnormal ectopic fat deposition and insulin resistance [13]. Recent studies showed, that resting adenosine triphosphate (ATP) production in the skeletal muscle of insulin-resistant patients is drastically decreased when compared with insulin-sensitive individuals, which clearly suggests the contribution of mitochondrial failure in insulin resistance development [14]. Moreover, the deteriorated function, number, and density as well as a decrease in rates of oxidative phosphorylation in muscle mitochondria in insulin-resistant Type 2 diabetes patients were correlated with an increase in the intramyocellular lipid concentration, and oxidative stress [15]. Mitochondrial malfunction is inextricably linked with insulin resistance since they are a main source of reactive oxygen species (ROS) and nitric oxide (NO) that both trigger adipocytes’ resistance to insulin and glucose uptake deterioration. Excessive generation of ROS and NO reduces mitochondrial metabolism and dynamics enhanced by elevated hyperglycemia as well as obesity or T2D [16]. Moreover, mitochondrial ROS attenuates insulin activity in adipocytes and inhibits insulin-stimulated GLUT4 translocation by degrading insulin-mediated redistribution of IRS-1 and PI3-kinas. Therefore, targeting mitochondrial metabolism and dynamics in insulin-resistant adipocytes has emerged as an important approach for increasing insulin sensitivity [17].

In recent years, accumulating data have demonstrated that sex hormone-binding globulin (SHBG) plays many functions besides being a passive career of steroid hormones that include active regulation of testosterone signaling and regulation of local expression of androgen-binding in adipose tissue [18]. Circulating SHBG levels are strongly associated with metabolic and hormonal factors, and their systemic level has been found to be decreased in patients with obesity, type second diabetes, or metabolic syndrome [19]. SHBG was also sowed to play an important role in activating insulin receptor activity and therefore increasing insulin sensitivity suggesting that SHBG may have a broader clinical utility in T2D or metabolic syndrome treatment [20]. Besides changes in both SHBG and PPARγ availability seem to be implicated in the pathogenesis of adipose tissue metabolic dysfunctions, whether both mediators’ crosstalk in the modulation of cellular homeostasis, remains still not elucidated. Therefore, this study aimed at evaluating the possible impact of SHBG on PPARγ activity in adipose-derived stromal cells (ASCs). We used for that a model of equine ASCs in which PPARγ has been silenced using siRNA transfection, and investigated the outcomes of SHBG treatment in terms of cell viability, apoptosis, premature senescence and mitochondrial functions to determine whether SHBG may represent a potential PPARγ mimic for future clinical application in the management of metabolic disorders.

## Materials and methods

All cell culture reagents and chemicals used in the study were obtained from Sigma Aldrich (Poznań, Poland), unless otherwise specified.

### Equine adipose-derived stromal (ASCs) cell culture

The equine ASCs utilized in the study were obtained from the Department of Experimental Biology at the University of Environmental and Life Sciences in Wroclaw, Poland. The cells used for this experiment were at passage 4 (p4). The ASCs cultures were maintained under standard conditions, which included Dulbecco’s modified Eagle’s medium Low Glucose (DMEM Low Glucose, 1000 mg/l glucose) supplemented with 10% foetal bovine serum (FBS), and 1% penicillin–streptomycin (PS) solution. The cells were cultured at constant and aseptic conditions in a CO_2_ incubator at 37 °C, and 95% humidity. The medium was changed every 2–3 days and cells were passaged when reached 80% confluence.

### Peroxisome proliferator-activated receptor gamma (PPARɣ) silencing and sex hormone binding globulin (SHBG) treatment

The PPARɣ gene expression was knockdown with the use of small interfering RNA (Silencer® Select Pre-designed siRNA, Ambion, siRNA ID: s10888) and Lipofectamine RNAiMAX Reagent (Invitrogen Life Technologies, Warsaw, Poland) in adherence to the instructions provided by the manufacturer. Prior to the experiment, the optimal siRNA concentration was chosen based on a screening test and a concentration of 20 nM was selected. The ASC cells were seeded onto 24 and 6-well plates in densities of 3 × 10^4^ and 1.5 × 10^5^ respectively per well and silencing was initiated when cultures reached 70% confluence. The PPARɣ gene expression was suppressed for a duration of 72 h. Subsequently, the medium was changed to DMEM Low Glucose medium (1000 mg/l glucose) supplemented with 0.02% bovine serum albumin (BSA), and 1% PS solution.

For the purpose of the experiment, three groups were established as follows: (1) HE ASCs (control cells), (2) HE ASCs PPARɣ^−^ (cells with silenced PPARɣ expression), (3) HE ASCs PPARɣ^−^ + SHBG 50 nM (cells with suppressed PPARɣ expression supplemented with SHBG protein at a concentration of 50 nM). The incubation with SHBG lasted for 24 h and then the cells were made ready for analysis by following the procedures specified for each experiment.

### Verification of PPARɣ silencing efficiency

To confirm the efficiency of PPARɣ gene silencing, a screening test was conducted, including two variants of siRNA (Silencer® Select Pre-designed siRNA, Ambion, siRNA ID: s10888 and s10887) at three concentrations (10, 15, and 20 nM). The silencing procedure was carried out as described above in "Materials and Methods". Suppression of PPARɣ gene expression was confirmed by western blot, Real-Time (RT) qPCR, and agarose gel electrophoresis analysis. RT-qPCR and western blot procedures are described below in Sects. 8 and 9, respectively. To evaluate the intensity of the PCR products, a 2% agarose gel was prepared by dissolving agarose powder in 1 × Tris/Borate/EDTA (TBE) buffer in a microwave oven. After boiling, SYBR™ Safe DNA Gel Stain (Invitrogen Life Technologies, Warsaw, Poland) was added. Once the gel polymerized, the PCR products (10 µL) and DNA Marker Ladder (MR20, Blirt) were mixed with DNA Gel Loading Dye (6X) (Invitrogen Life Technologies, Warsaw, Poland) and loaded into the gel. Electrophoresis was conducted at a voltage of 100 V for a duration of 60 min. The ChemiDoc MP imaging system (Bio-Rad, Hercules, CA, USA) was used for gel visualisation, while the quantification was carried out using the Image Lab software (Bio-Rad, Hercules, CA, USA).

### Analysis of cell viability and oxidative stress by capillary flow cytometry

Analysis of viability and oxidative stress detection were assessed using capillary flow cytometry (Muse® Cell Analyzer, Merck Millipore).

The apoptosis profile was established using the MUSE Annexin V & Dead Cell Kit (Luminex Corporation) following the manufacturer's instructions. The cells were detached using trypsin, centrifuged, and diluted in PBS containing 1% FBS. A cell suspension containing 1 × 10^5^ cells was then mixed with 100 µl Annexin V/dead reagent and incubated for 20 min at room temperature in the dark. Subsequently, the cells were analyzed using the Muse Cell Analyzer. The distribution of apoptotic cells was assessed by identifying four populations: (1) nonapoptotic cells [Annexin V( −), 7-AAD( −)]; (2) early apoptotic cells [Annexin V( +), 7-AAD( −)]; (3) late apoptotic cells [Annexin V( +), 7-AAD( +)] 4) dead cells [Annexin V( −) and 7-AAD( +)].

The analysis of oxidative stress was conducted using the Muse Oxidative Stress Kit (Luminex Corporation). After trypsinization, the obtained cell suspension (1 × 10^5^) was added to the Muse Oxidative Stress working solution reagent (200 µl) prepared according to the manufacturer’s protocol. The obtained suspension was incubated at 37 °C for 30 min and then the samples were read using the MUSE mini flow cytometer. The assessment of the percentage of cells experiencing oxidative stress through intracellular detection of superoxide radicals involved the identification of two distinct populations: (1) Negative cells [ROS–] and (2) cells with ROS activity [ROS +].

### Evaluation of cellular senescence with β-galactosidase staining

The analysis of cellular senescence was conducted by utilizing the Senescence Cells Histochemical Staining Kit, following the instructions provided by the manufacturer. After the PPARɣ silencing and SHBG treatment, the cells were fixed and incubated with the staining mixture for a duration of 24 h at 37 °C without CO_2_. Microphotographs were captured at magnifications of × 100 and × 400 using the Leica DMi1 inverted microscope (Leica Microsystems). To calculate the percentage of cells expressing β-galactosidase (senescent cells), the Fiji ImageJ software and Colour Pixel Counter plugin (version 1.52n, Wayne Rasband, National Institutes of Health, USA) were used.

### Assessment of mitochondrial transmembrane potential

In order to monitor mitochondrial health, the membrane-permeant MitoProbeTM JC-1 Assay Kit for Flow Cytometry (Life Technologies) has been used according to the manufacturer’s instructions. Briefly, 10^6^ cells were dissolved in 1 ml of PBS, and then 10 µL of 200 µM JC-1 (final concentration of 2 µM) was added. The cells were incubated at 37 °C, 5% CO_2_, for 30 min. For the control sample, 1 µL of 50 mM CCCP (supplied with the kit, final concentration of 50 µM) was added, and the cells were incubated at 37 °C for 5 min. Then the cells were suspended in 500 µl of PBS and subjected to analysis using the BD LSRFortessa™ Cell Analyzer (Becton Dickinson, Franklin Lakes, New Jersey, USA). Additionally, active mitochondria have been examined through confocal microscopy. For this purpose, the cells were plated onto microscope glass coverslips positioned within 24-well plates. Following the experiment, the cells were stained as described above and then were fixed with 4% paraformaldehyde (PFA) at room temperature for 30 min. Next, slides were prepared using ProLong™ Diamond Antifade Mounting medium with DAPI (Invitrogen Life Technologies, Warsaw, Poland). Subsequently, images were captured using a confocal microscope at a magnification of × 630. (Leica DMi8, Leica Microsystems, KAWA.SKA Sp. z o.o., Poland).

### Analysis of mitochondrial network development

To analyze the mitochondrial network, MitoRed fluorescent dye was used. The dye was diluted at a ratio of 1:1000 in the complete growth culture medium and applied for 30 min at 37 °C in a CO_2_ incubator. Following the staining, the cells were fixed with 4% PFA at room temperature for 30 min. Nuclei staining was performed using the ProLong™ Diamond Antifade Mountant containing DAPI. The cells were then observed using a confocal microscope at a magnification of × 1000 (Leica DMi8, Leica Microsystems, KAWA.SKA Sp. z o.o., Poland). The MicroP software was employed to evaluate the morphology of the mitochondria. Furthermore, the intensity of mitochondrial staining was analyzed by the Fiji ImageJ software with the assistance of the 'Colour Pixel Counter’ plugin.

### Determination of relative gene expression using qRT-PCR

Following the experimental treatment procedures, the expression of selected markers involved in apoptosis and mitochondrial biogenesis was assessed. To isolate total RNA, 1 mL of Extrazol® (Blirt DNA, Gdansk, Poland) was used according to the manufacturer’s instructions. The obtained RNA was diluted in DEPC-treated water. The quantity and purity of the RNA were determined spectrophotometrically at 260 and 280 nm wavelengths using an Epoch spectrophotometer (Biotek, Germany). The isolated RNA underwent purification with DNAse I digestion prior to reverse transcription, using 500 ng of total RNA and the PrecisionDNAse kit (PrimerDesign, BLIRT S.A. Gdansk, Poland). Reverse transcription was conducted using the Tetro cDNA Synthesis Kit (Bioline Reagents Limited, London, UK) following the provided protocol. The process of DNA digestion and cDNA synthesis was performed in a T100 Thermal Cycler (Bio-Rad, Hercules, CA, USA). The cDNA matrices were utilized for RT-qPCR analysis using the SensiFAST SYBR®&Fluorescein Kit (Bioline Reagents Ltd., London, UK). Each reaction had a final volume of 10 µl, consisting of 1 µl of cDNA, 5 µl of Master Mix, and the primer concentration set at 500 nM. Quantitative PCR was performed using the CFX Connect Real-Time PCR Detection System. (Bio-Rad, Hercules, CA, USA). Reaction conditions were as follows: an initial denaturation step at 95 °C, 2 min, followed by 40 cycles of denaturation at 95 °C, 15 s, annealing (specific primer temperature), 15 s, and elongation at 72 °C, 15 s. The results of the analysis were reported based on the expression of Glyceraldehyde-3-phosphate dehydrogenase (GAPDH). Gene expression levels were determined relative to a reference using the RQMAX method, and the results were presented on a logarithmic scale. The primer sequences used in the analysis are provided in Table 1.

**Table 1:** List of primers used in the RT-qPCR analysis

**Gene**	**Primer**	**Sequence 5′-3′**	**Amplicon lenght (bp)**	**Annealing temperature (°C)**	**Accession no**
*PPARy*	F:	TTTCGCTCAGTGGAAGCTGT	138	62	XM_023619952.1
	R:	GGAGGCCAGCATGGTGTAAA			
*Cat*	F:	ACCAAGGTTTGGCCTCACAA	112	62	XM_014729341.2
	R:	TTGGGTCAAAGGCCAACTGT			
*Gpx*	F:	TCGAGCCCAACTTCACACTC	178	62	NM_001166479.1
	R:	AAGTTCCAGGCGACATCGTT			
*Sod1*	F:	CATTCCATCATTGGCCGCAC	130	62	NM_001081826.3
	R:	GAGCGATCCCAATCACACCA			
*Sod2*	F:	GGACAAACCTGAGCCCCAAT	125	62	NM_001082517.2
	R:	TTGGACACCAGCCGATACAG			
*NRF2*	F:	AAACCAGTGGGTCTGCCAA	188	62	XM_001496992.5
	R:	AATGAAGTCTGGGCTCTCGATG			
*Mfn*	F:	AAGTGGCATTTTTCGGCAGG	217	62	XM_023623336.1
	R:	TCCATATGAAGGGCATGGGC			
*Mief-1*	F:	ATGCTGGGCATCGCTACAC	284	62	XM_023631522.1
	R:	CGGAGCCGTGACTTCTTCAA			
*Mief-2*	F:	CGTTCTATTATCAGGCAGGTCC	108	62	XM_005597824.3
	R:	AGAACTCTGCCATGGTCTTCT			
*Miro1*	F:	GATCCTGCTGGTGGGAGAAC	88	62	XM_023651639.1
	R:	GGGAGGAACCTCTTCTGGGA			
*mTOR*	F:	GGGCAGCATTAGAGACGGTG	221	62	XM_005607536.3
	R:	ATGGTTGATTCGGTGTCGCA			
*Pgc1a*	F:	GGCCTTCTAAACGTGGGACA	135	62	NM_001242553.1
	R:	CCGGAGGTCTGCCATTTTCT			
*Pink*	F:	GCACAATGAGCCAGGAGCTA	115	54,6	XM_023635588.1
	R:	GGGGTATTCACGCGAAGGTA			
*Parkin*	F:	CTGGAGGATTTAGTCCCGGAGC	138	54,6	XM_005608126.3
	R:	CCATGGCTGGAGTTGAACCTG			
*OGG*	F:	GTGCCCGAGCTATCTTGGAA	218	62	XM_023620020.1
	R:	GTAGTCACGCTGGGCAATCT			
*MUTYH*	F:	AACTGCAGAATTGGGCTGGG	277	62	XM_023631088.1
	R:	CTAGACGGGGTGGCTCTTCT			
*POLG*	F:	CACAACGTCTGCTTTGACCG	289	62	XM_023650178.1
	R:	CTCCCACATAGAGGCTGTGC			
*Gapdh*	F:	GATGCCCCAATGTTTGTGA	250	54,6–62	NM_001163856.1
	R:	AAGCAGGGATGATGTTCTGG			

### Analysis of protein profiles with western blotting

Following the cell culturing process under the defined experimental conditions, the cells were lysed using ice-cold RIPA buffer supplemented with 1% proteases and phosphatases inhibitors cocktail. The protein concentration of the samples was determined with the Bicinchoninic Acid Assay Kit. Then, a final concentration of 10 µg proteins was diluted in a 4X Laemmli Loading Buffer (Bio-Rad, Hercules, CA, USA) and subjected to denaturation at a temperature of 95 °C for 5 min. Subsequently, the samples and BLUeye Prestained Protein Ladder were separated onto a 10 or 12% sodium dodecyl sulfate–polyacrylamide gel electrophoresis (SDS-PAGE) at 100 V for 90 min. After the electrophoresis step, the proteins were transferred onto a PVDF membrane using 1 × Tris-base/Glycine buffer and the Mini Trans-Blot® system (Bio-Rad, Hercules, CA, USA) for 60 min at 100 V. After the transfer process, the membranes were subjected to a blocking step for 1 h using a 5% skim milk solution prepared in Tris/NaCl/Tween buffer (TBST). The membranes were afterwards incubated overnight at 4 °C with specific primary antibodies as indicated in Table 2. Subsequently, the membranes were washed 5 times for 5 min each with TBST buffer. After the washing step, the membranes were incubated with an HRP-conjugated secondary antibody for 60 min at room temperature. All antibodies were prepared in 5% skim milk in TBST buffer. The membranes were then washed as described above and analyzed using the Bio-Rad ChemiDoc™ XRS system using Clarity Western ECL Substrate (Bio-Rad, Hercules, CA, USA). The signal intensity was quantified using the Image Lab™ Software (Bio-Rad).

**Table 2:** The antibodies used for Western Blot

**Antibody**	**Catalog No**	**Dilution**
PARKIN	Novus Biologicals, nbp2-67,017	1:250
MFN	Biobyrt, orb11040	1:500
PINK	Biorbyt, orb331223	1:250
PPARG	Aviva Systems Biology, ARP32880	1:500
HIF-1A	Aviva Systems Biology, ARP30572_P050	1:500
β-Actin	Sigma Aldrich, a2066	1:5000
Goat Anti-Rabbit IgG H&L (HRP)	Abcam, ab205718-500	1:10000
Rabbit Anti-Mouse IgG H&L (HRP)	Abcam, ab6728-1	1:10000

### Statistical analyses

Statistical analysis was conducted using GraphPad Prism 8 (GraphPad Software, San Diego, CA, USA) with the application of one-way analysis of variance (ANOVA) followed by Tukey’s post-hoc test. Each analysis was performed with a minimum of three replicates. Statistical significance was determined at a *p*-value of less than 0.05. Significance levels were denoted by asterisks: *p < 0.05, **p < 0.01, ***p < 0.001, and ****p < 0.0001. Non-significant differences were indicated as “ns.”

## Results

### PPARγ silencing efficiency

**Fig. 1:**
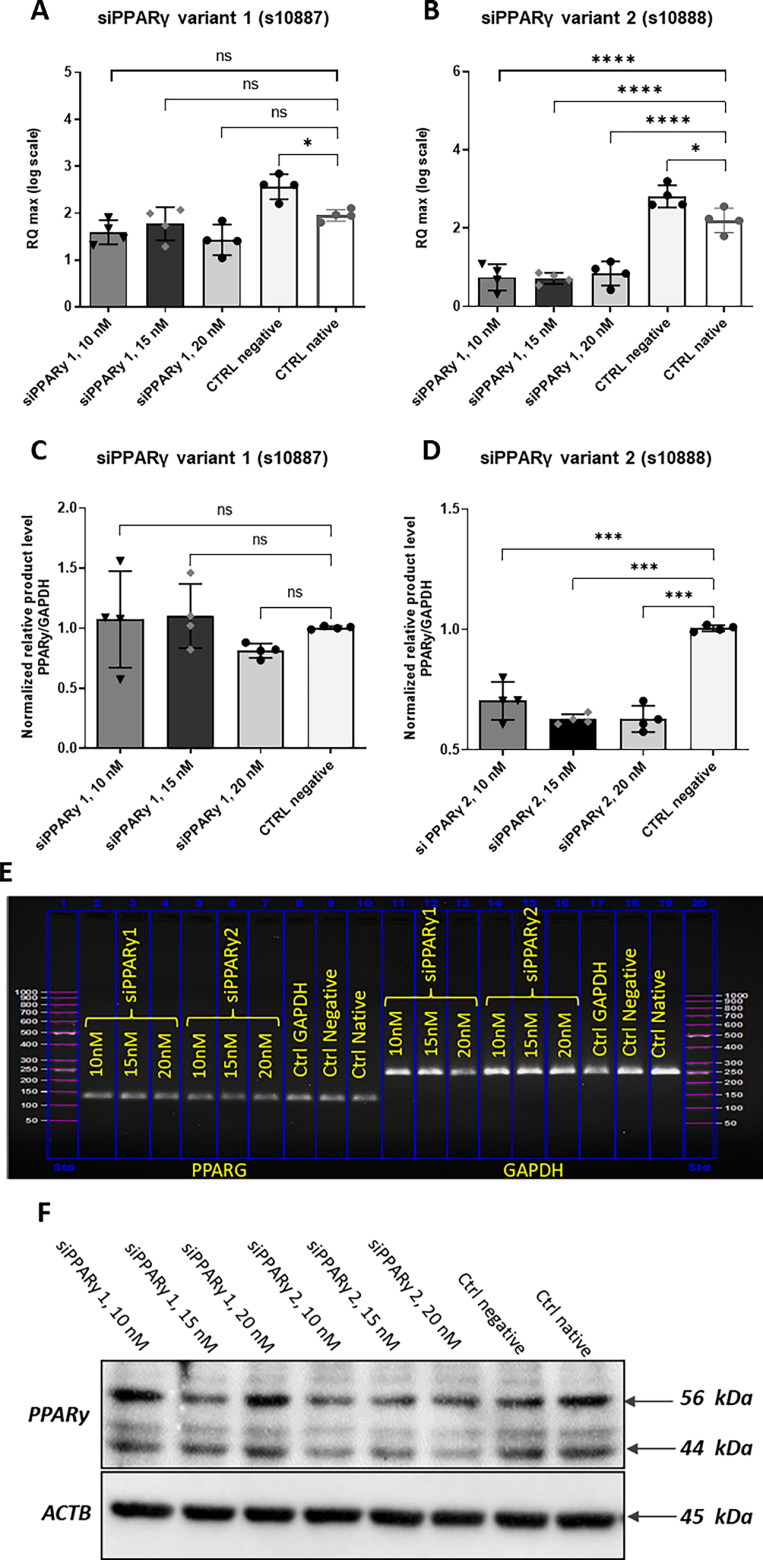
PPARγ silencing efficiency. **A** Relative gene expression of PPARG after silencing with siRNAs variant 1 s10887. **B** Relative gene expression of PPARG after silencing with siPPARγ variant 2 s10888. **C** Relative qPCR amplicon abundance of PPARG following silencing with siRNA variant 1 s10887 normalized to GAPDH. **D** Relative qPCR amplicon levels of PPARG following silencing with siRNA variant 2 s10888 normalized to GAPDH. **E** Agarose gel photograph showing PCR product of both siPPARγ variant 1 and variant 2 at various concentrations and GAPDH. **F** Representative immunoblots showing the protein levels of PPARγ under two silencing conditions and internal β-actin control. Results are expressed as mean ± SD. Statistical significance is marked with asterisks (*); *p < 0.05, **p < 0.01, ***p < 0.001, ****p < 0.0001

In order to lower the expression of the PPARγ gene, two siPPARγ variants have been designed and tested for their knockdown efficiency at the mRNA and protein level. As shown in Fig. 1B, D, the incubation of EqASCs with the siPPARγ variant n°2 resulted in a significant downregulation of PPARγ mRNA (*p* < *0.001*) at the three tested concentrations, by contrast to the variant n°1 which did not induce any changes in the levels of PPARγ transcripts when compared to the native control (Fig. 1A, C). Similar trends were observed at the protein level, where siPPARγ variant 2 was found as being the most effective in lowering PPARγ protein amount (Fig. 1F).

### Impact of PPARγ silencing and SHBG treatment on cell viability and senescence

The initiation of apoptosis was assessed after 72 h of PPARγ silencing and a further 24 h of incubation with 50 nM SHGB. Apoptotic and necrotic cells were detected by capillary flow cytometry using a MUSE cell analyzer with annexin V and 7-AAD double staining kit.

**Fig. 2:**
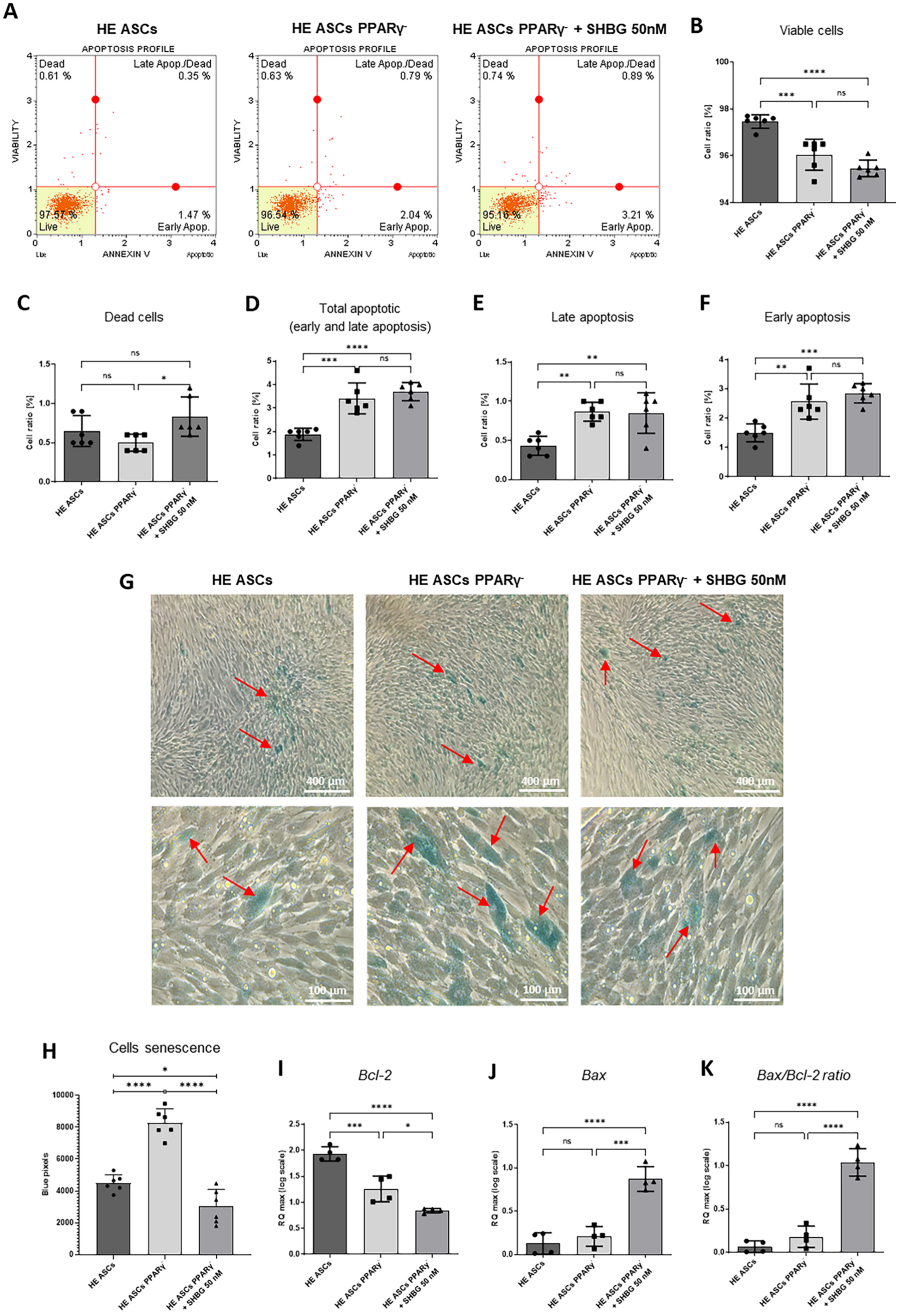
Comparison of apoptosis and necrosis in healthy adipose-derived stromal cells (ASCs), PPARγ-silenced ASCs, and PPARγ-silenced ASCs after incubation with 50 nM SHBG. **A** Representative cytograms from evaluating apoptosis and cell death with the Muse Cell Analyzer. **B**–**F** Percentage of viable, apoptotic, apoptotic/dead and dead cells assessed using Annexin/7-AAD apoptosis kit. **G** Representative images of β-galactosidase staining. Positive blue staining with β-galactosidase is indicated by arrows. Scale bar: 50 µm. **H** Cells senescence — number of pixels stained with β-galactosidase (blue) with *n* = 3. **I**–**K** Apoptosis-associated gene transcript levels determined by qRT-PCR: Bcl-2 and Bax, and their ratio as the pro-apoptotic index; results are expressed as mean ± SD. Statistical significance is marked with asterisks (*); *p < 0.05, **p < 0.01, ***p < 0.001

As shown in Fig. 2, both silencing of the PPARγ gene alone and in combination with incubation with 50 nM SHBG resulted in a statistically significant decrease in cell viability compared to the native control, with a greater effect on cells additionally incubated with SHBG protein, but the effect of adding SHBG was not statistically significant (Fig. 2A, B). The number of cells in the early phase of apoptosis increased by approximately 0.6% in the case of PPARγ gene silencing and by approximately 1.7% after additional incubation with 50 nM SHBG (Fig. 2F). Silencing of the PPARγ gene as well as subsequent incubation with 50 nM SHBG showed a statistically significant pro-apoptotic effect, increasing the number of cells both in the early and late apoptosis phase (Fig. 2D, E, F). Additional incubation with 50 nM SHBG did not enhance the pro-apoptotic effect compared to PPARγ-silenced cells. The number of cells in the necrotic phase was not statistically significantly different compared to the native control for both SHBG administration and only PPARγ gene silencing (Fig. 2C). However, a statistically significant increase in dead cell number was observed in cells with the PPARγ gene silenced after the administration of the SHBG protein.

The level of β-galactosidase was assessed by histochemical staining. Example microphotographs of positively stained cells (blue staining) are shown in Fig. 2G. The photos taken were analyzed by counting the number of pixels on the microphotographs using the Fiji ImageJ open-source platform (Fig. 2H). Based on the calculations, it can be concluded that as a result of silencing the PPARγ gene, the level of β-galactosidase was statistically significantly higher compared to the native group, as well as in the case of additional incubation with SHBG. At the same time, the level of β-galactosidase was statistically significantly lower compared to the native group.

Due to the significant effect of silencing the PPARy gene and incubation with SHBG on the number of cells in the apoptotic phase, the level of Bax and Bcl-2 proteins was assessed. Bax and Bcl-2 affect the regulation of apoptosis by controlling the flow of ions (K + , H + , Cl − , Ca2 +) and reactive oxygen species (ROS) in the cell, the release of apoptogenic factors from the mitochondria, such as AIF, cytochrome c and activation of caspases and DNase. Bcl-2 has an anti-apoptotic effect, its level was statistically significantly reduced in PPARy-silenced cells compared to the native control (Fig. 2I). The group treated with 50 nM SHBG had statistically significantly lower Bcl-2 level compared to the other two groups. The Bcl-2 protein prevents the oligomerization of Bax, which accelerates apoptosis by increasing the permeability of the outer membrane of the mitochondria by creating pores. A statistically significant increase in Bax level was observed in the SHBG-treated group compared to the native group and PPARy-silenced ASCs (Fig. 2J). A good representation of the obtained results is the pro-apoptotic index, i.e., the Bax/Bcl-2 ratio. A statistically significant increase in the Bax/Bcl-2 ratio after incubation with 50 nM SHBG compared to the native group and PPARy-silenced ASCs group was observed in the experiment (Fig. 2K).

### Status of oxidative stress and endogenous antioxidant enzymes expression in PPARγ-silenced and SHBG-treated cells

**Fig. 3:**
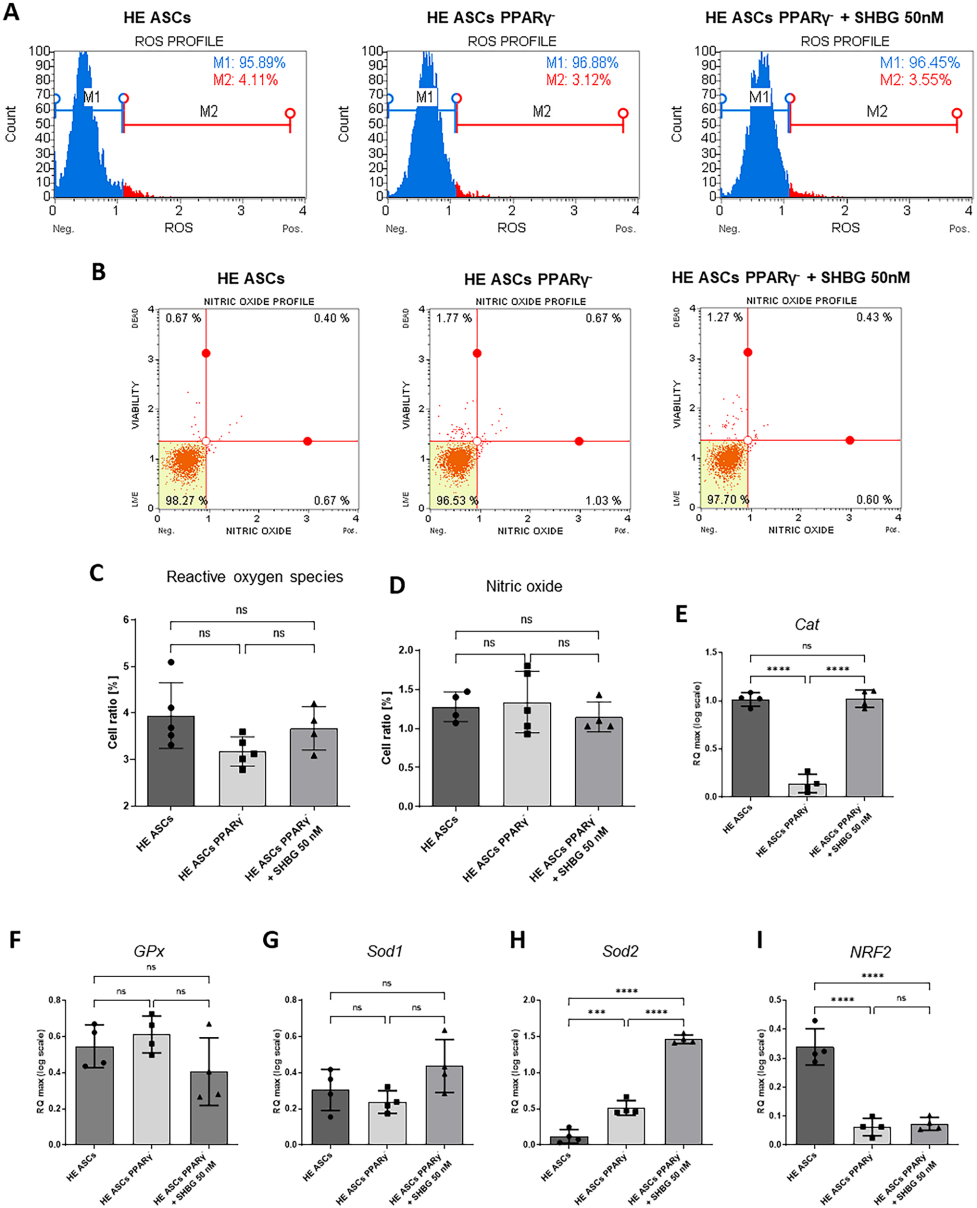
Changes in extracellular oxidative stress in healthy adipose-derived stromal cells (ASCs), PPARγ-silenced ASCs, and PPARγ-silenced ASCs after incubation with 50 nM SHBG. **A** Representative cytograms from the measurement of free oxygen radicals (ROS). **B** Representative cytograms from the analysis of nitric oxide (NO) levels. **C** Oxygen free radicals measured with a MUSE capillary flow cytometer. **D** Released nitric oxide (NO). **E**–**I** Relative gene expression of key antioxidant factors catalase (Cat), superoxide dismutase (SOD1/SOD2), glutathione peroxidase (GPx), and transcription factor Nrf2; results are expressed as mean ± SD. Statistical significance is marked with an asterisk (*): *p < 0.05, **p < 0.01, and ***p < 0.001

The effect of SHBG on intracellular ROS production has been investigated using Muse Oxidative Stress Kit (Fig. 3A). The obtained flow cytometry results showed that neither PPARγ gene silencing nor 50 nM SHBG administration significantly affected the percentage of cells undergoing oxidative stress (Fig. 3C).

Nitric oxide levels were assessed using a ready-to-use kit and MUSE cell analyzer. There were no statistically significant differences between the total concentrations of nitric oxide in the study groups (Fig. 3B, D).

Cells are protected against oxidative stress by an antioxidant system consisting, among others, of enzymes such as catalase (CAT), superoxide dismutase (SOD), and glutathione peroxidase (GPx). In addition, the transcription factor Nrf2 plays a key role in the protective process against the harmful effects of oxidative stress. Comparing mRNA levels, neither silencing of the PPARγ gene nor subsequent administration of 50 nM SHBG did not cause a significant change in the activity of glutathione peroxidase or superoxide dismutase 1 (SOD1) (Fig. 3F, G). However, a statistically significant increase in SOD2 activity was observed in PPARγ-silenced cells (Fig. 3H). Moreover, incubation for 24 h with 50 nM SHBG resulted in an even greater increase in SOD2 which was statistically significant compared to both native control and PPARγ-silenced cells. Comparing the mRNA level of the transcription factor NRF2, a statistically significant decrease was observed after silencing the PPARγ gene (Fig. 3I). At the same time, 24 h of incubation with 50 nM SHBG did not affect the NRF2 level which remained at a similar level as in cells with silenced PPARγ gene. Interestingly, PPAR depletion induced a visible downregulation of CAT transcript when compared to native cells. Subsequently application of exogenous SHBG enabled to restore the mRNA levels of CAT to a basal level comparable to that of native cells (Fig. 3E).

### Influence of PPARγ knockdown and SHBG treatment on mitochondrial metabolism

**Fig. 4:**
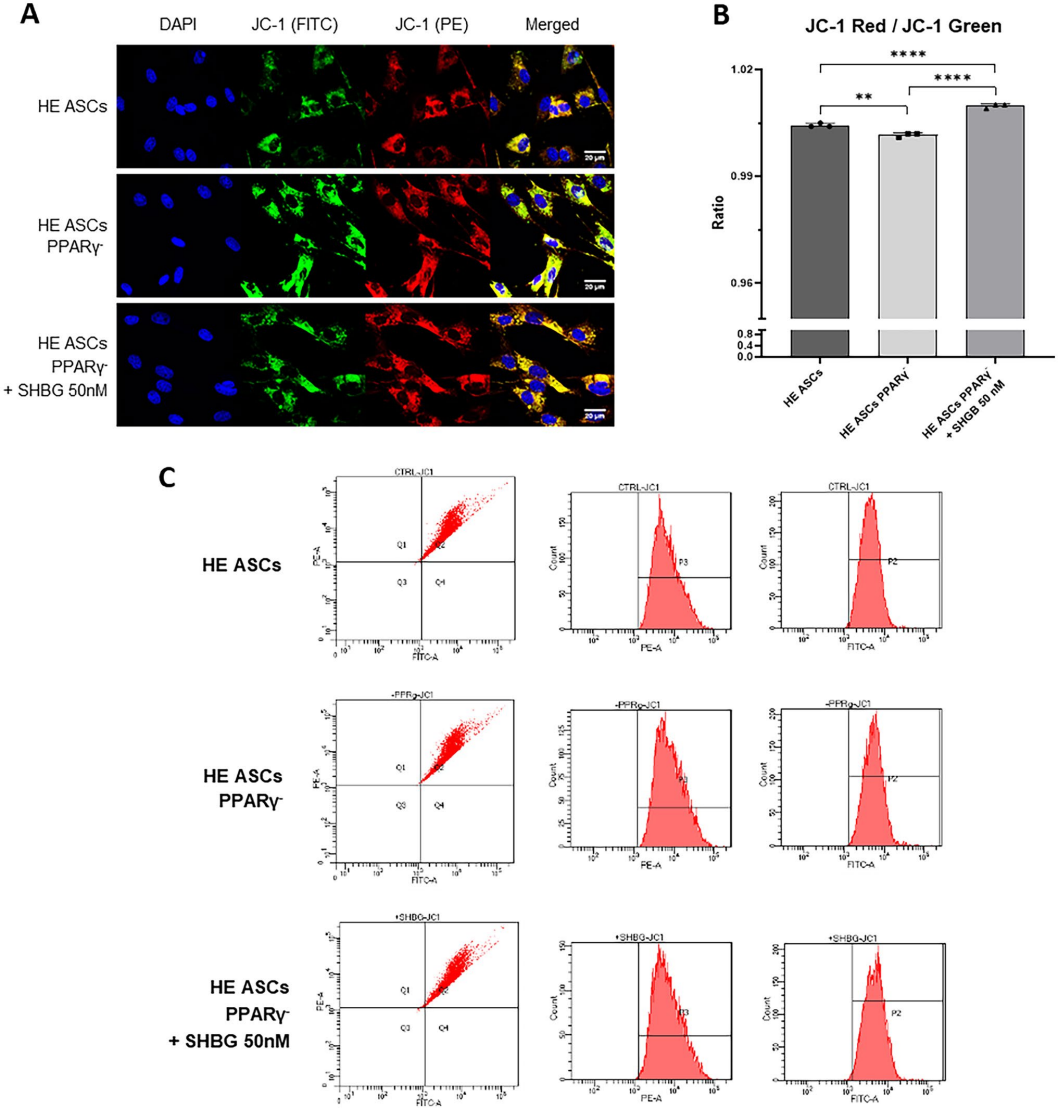
Mitochondrial membrane potential in healthy adipose-derived stromal cells (ASCs), PPARγ-silenced ASCs, and PPARγ-silenced ASCs after incubation with 50 nM SHBG. **A** Representative visualization images of mitochondria in ASCs with JC-1 FITC (green), JC-1 PE (red), and DAPI staining (blue) using confocal microscopy. Scale bar: 20 µm. **B** Mitochondrial membrane potential estimation by JC-1 Red to JC-1 Green Ratio. **C** Mitopotential analysis using the BD LSRFortessa cell analyser; results are expressed as mean ± SD. Statistical significance is marked with asterisks (*): *p < 0.05, **p < 0.01

Mitochondrial membrane potential (MtMP) was assessed by staining with JC-1 dye. An evaluation was performed using BD LSRFortessa Cell Analyzer, and additionally, through confocal microscopy (Fig. 4A, C). The number of red/orange-stained mitochondria was increased in cells incubated with 50 nM SHBG compared to the native group as well as PPARγ-silenced cells. In contrast, the number of mitochondria with reduced MtMP (emitting green fluorescence) was higher in PPARγ-silenced cells than in the native group. There were significant differences in JC-1 red/JC-1 green ratios between the groups. This value was the lowest in PPARγ-silenced cells, higher in the native group, and the highest in PPARγ-silenced cells incubated with 50 nM SHBG (Fig. 4B).

**Fig. 5:**
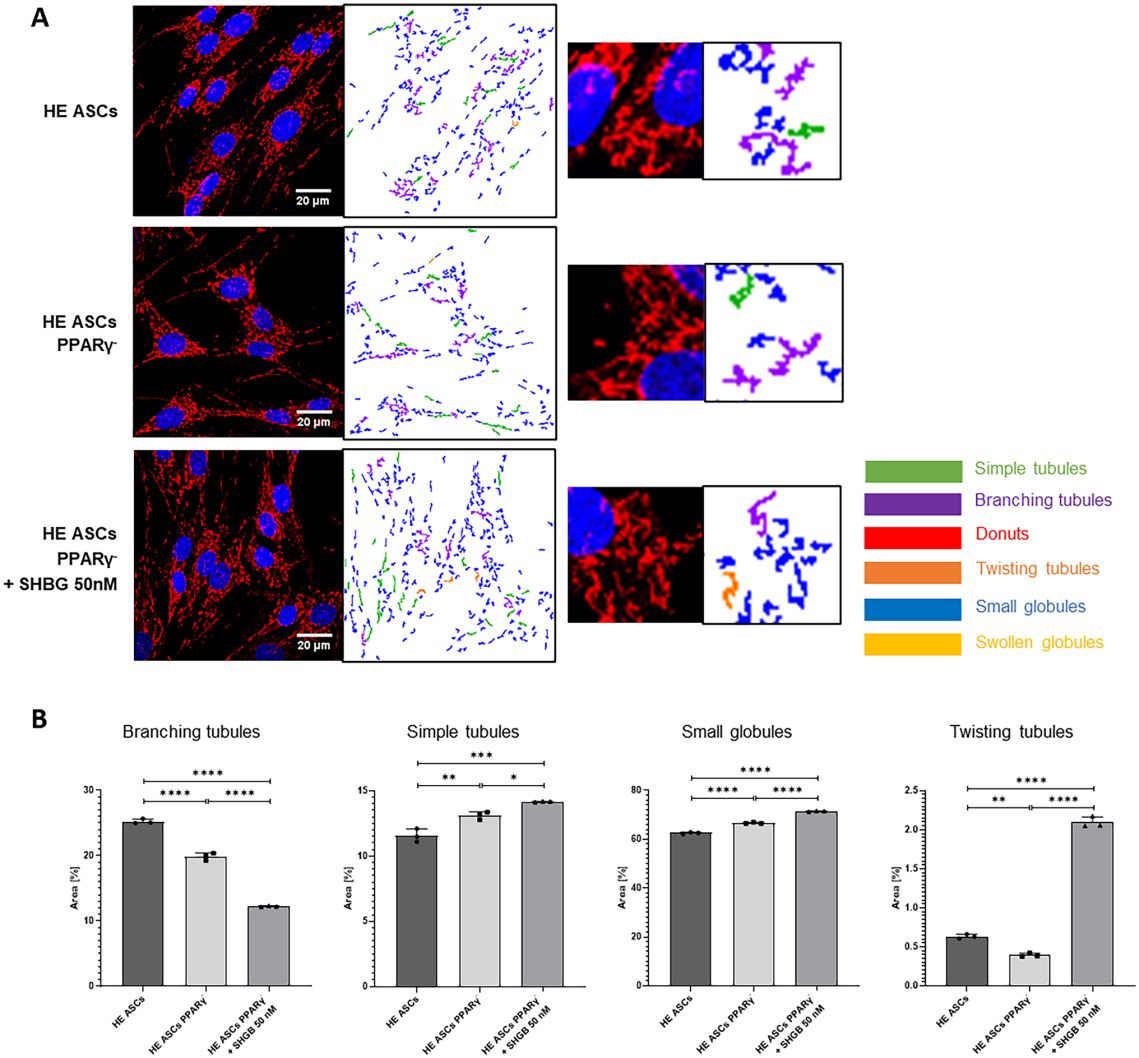
Average area of mitochondrial subtypes in individual cells in healthy adipose-derived stromal cells (ASCs), PPARγ-silenced ASCs, and PPARγ-silenced ASCs after incubation with 50 nM SHBG; the percentages shown in the legends are the ratio of cells in each population tested. Results are expressed as mean ± SD; statistical significance is marked with asterisks (*): *p < 0.05, **p < 0.01

Both PPARγ silencing and subsequent incubation with 50 nM SHBG significantly affected the dynamics of the mitochondrial network (Fig. 5). In native cells, mitochondria were characterized by highly branched morphology; after PPARγ silencing, mitochondria morphology shifts to a more fragmented structure composed of more simple tubules and small globules. The administration of 50 nM SHBG to PPARγ-silenced cells only increased this tendency in a statistically significant way. It is noteworthy that PPARγ silencing reduced the number of twisted tubules, while subsequent incubation with SHBG caused them to increase significantly to a much higher number than in the native group.

**Fig. 6:**
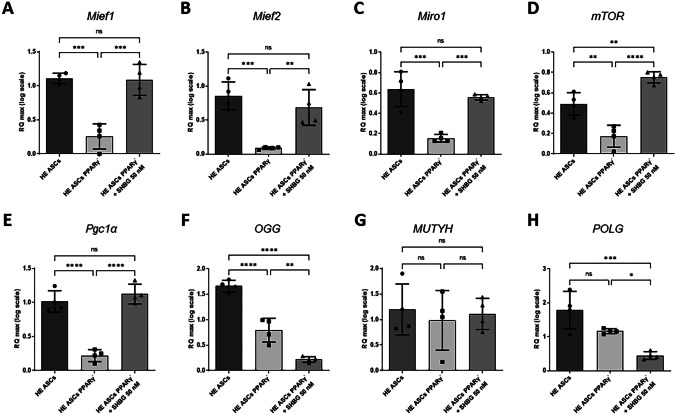
Changes in mRNA level of mitochondrial function markers (**A**–**E**) and markers associated with mtDNA damage (**F**–**H**) in healthy adipose-derived stromal cells (ASCs), PPARγ-silenced ASCs, and PPARγ-silenced ASCs after incubation with 50 nM SHBG; The levels of mRNA were evaluated using RT-qPCR. Results are expressed as mean ± SD. Statistical significance is marked with asterisks (*): *p < 0.05, **p < 0.01

mRNA levels were determined to assess mitochondrial function. Parameters such as Mfn, Mief1, Mief2, Miro1, mTOR, Pgclα, Pink, and Parkin were evaluated (Fig. 6A–H). Mitofusin (MFN) is involved in membrane binding and mitochondrial grouping. Mitochondrial elongation factor 1 (MIEF1) and mitochondrial elongation factor 2 (MIEF2) affect mitochondrial fission mediator dynamin-related protein 1 (Drp1). MIEF2 mRNA levels were generally lower than MIEF1 mRNA levels in all study groups. Miro is responsible for mitochondrial transport on microtubules by sensing both cellular and mitochondrial calcium ions. mTOR modulates mitochondrial function, by affecting mitochondrial energy production and regulating the processes of mitophagy. Pgclα is responsible for the induction of mitochondrial biogenesis and may increase the oxidation of fatty acids. A statistically significant decrease in the level of each of the assessed parameters was observed after silencing the PPARγ gene compared to native cells. In turn, as a result of incubation with 50 nM SHBG, a statistically significant increase in the level of mRNA was shown to a level similar to that in the group of native cells (Fig. 6A–C). In the case of mTOR, after administration of 50 nM SHBG, the mRNA level was significantly higher also compared to native cells (Fig. 6D). The level of *Pgc1α* mRNA appeared noticeably lower after PPARγ silencing, while in cells incubated with SHBG, its expression was found to be restored to basal level comparable to native control (Fig. 6E).

The mRNA levels of OGG, MUTYH, and POLG markers associated with mtDNA damage were also examined (Fig. 6F–H). OGG is involved in the DNA repair process, including mtDNA. A statistically significant reduction in mRNA level was observed after PPARγ silencing compared to the native group. In addition, subsequently, incubation with 50 nM SHBG caused an even greater reduction in OGG mRNA level. MUTYH is involved in DNA base excision repair and activates nuclear and mitochondrial cell death pathways. No differences in MUTYH mRNA levels were observed in the study. POLG is responsible for the replication of the mitochondrial genome. The measured POLG mRNA level was statistically significantly lower after incubation with 50 nM SHBG compared to both the native group and the group of PPARγ-silenced cells.

**Fig. 7:**
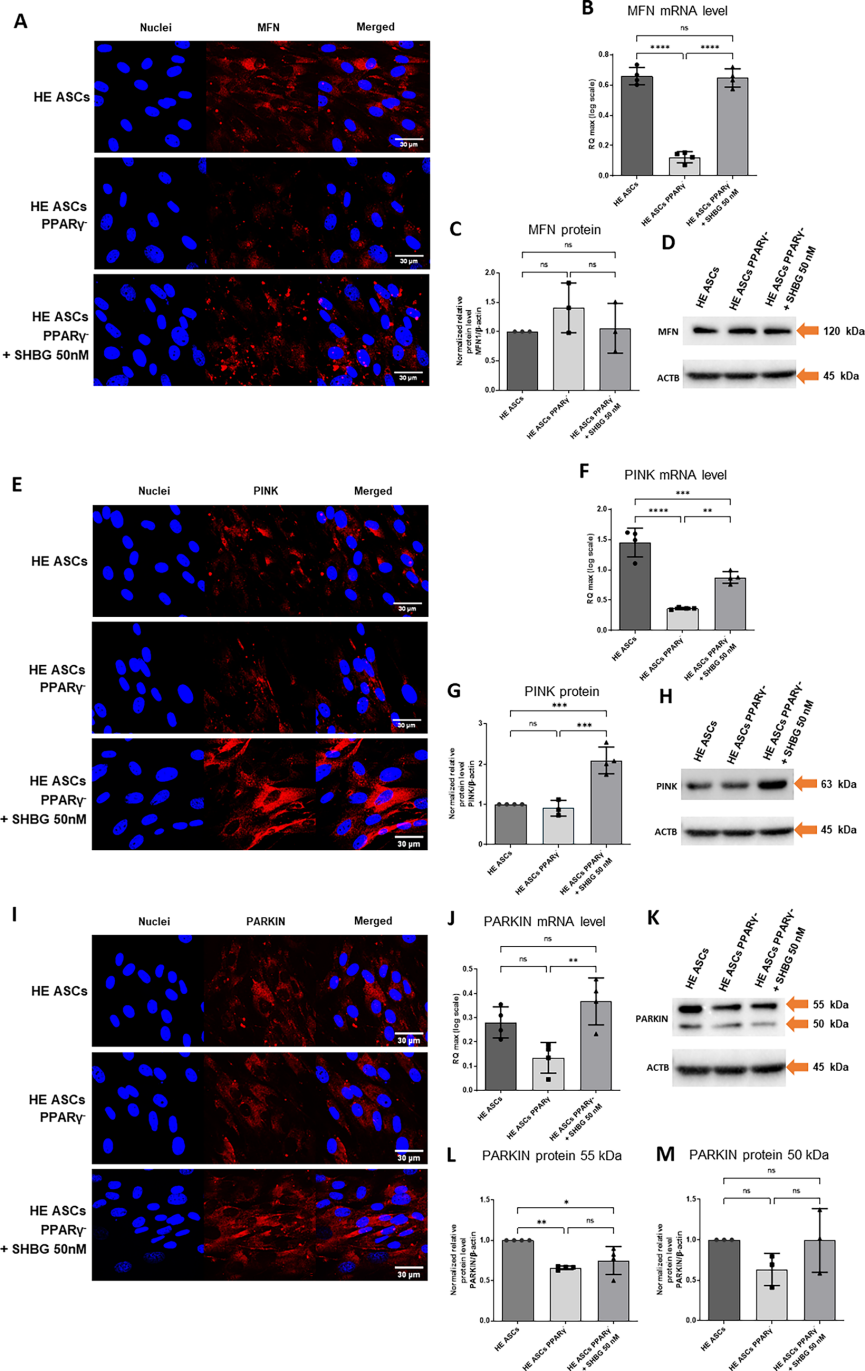
Changes in MFN, PINK1, and PARKIN markers levels in healthy adipose-derived stromal cells (ASCs), PPARγ-silenced ASCs, and PPARγ-silenced ASCs after incubation with 50 nM SHBG. **A**, **E**, **I** Intracellular localization of proteins by confocal microscopy. **B**, **F**, **J** mRNA levels determined by RT-qPCR. **C**, **G**, **L**, **M** protein levels calculated using Image Lab software after normalization to β-actin as an internal protein control. **D**, **H**, **K** Representative Western blots; results are expressed as mean ± SD. Statistical significance is marked with asterisks (*): *p < 0.05, **p < 0.01

Immunofluorescence staining was performed to visualize MFN, PINK, and PARKIN, and the effects were photographed using a confocal microscope (Fig. 7A, E, I). Western blot analysis showed that the apparent molecular weights of MFN1, PARKIN, and PINK were ∼120 kDa, ∼50–55 kDa, and ∼63 kDa (Fig. 7D, H, K), respectively, which is consistent with other reports [21]. The level of MFN1 protein did not differ significantly between all study groups (Fig. 7C). On the other hand, PINK protein level was statistically significantly higher in the group treated with 50 nM SHBG compared to the native group and PPARγ-silenced cells (Fig. 7G). In turn, the level of PARKIN protein was statistically significantly higher in native cells compared to both other groups — cells with silenced PPARγ gene and cells subsequently incubated with 50 nM SHBG (Fig. 7L).

**Fig. 8:**
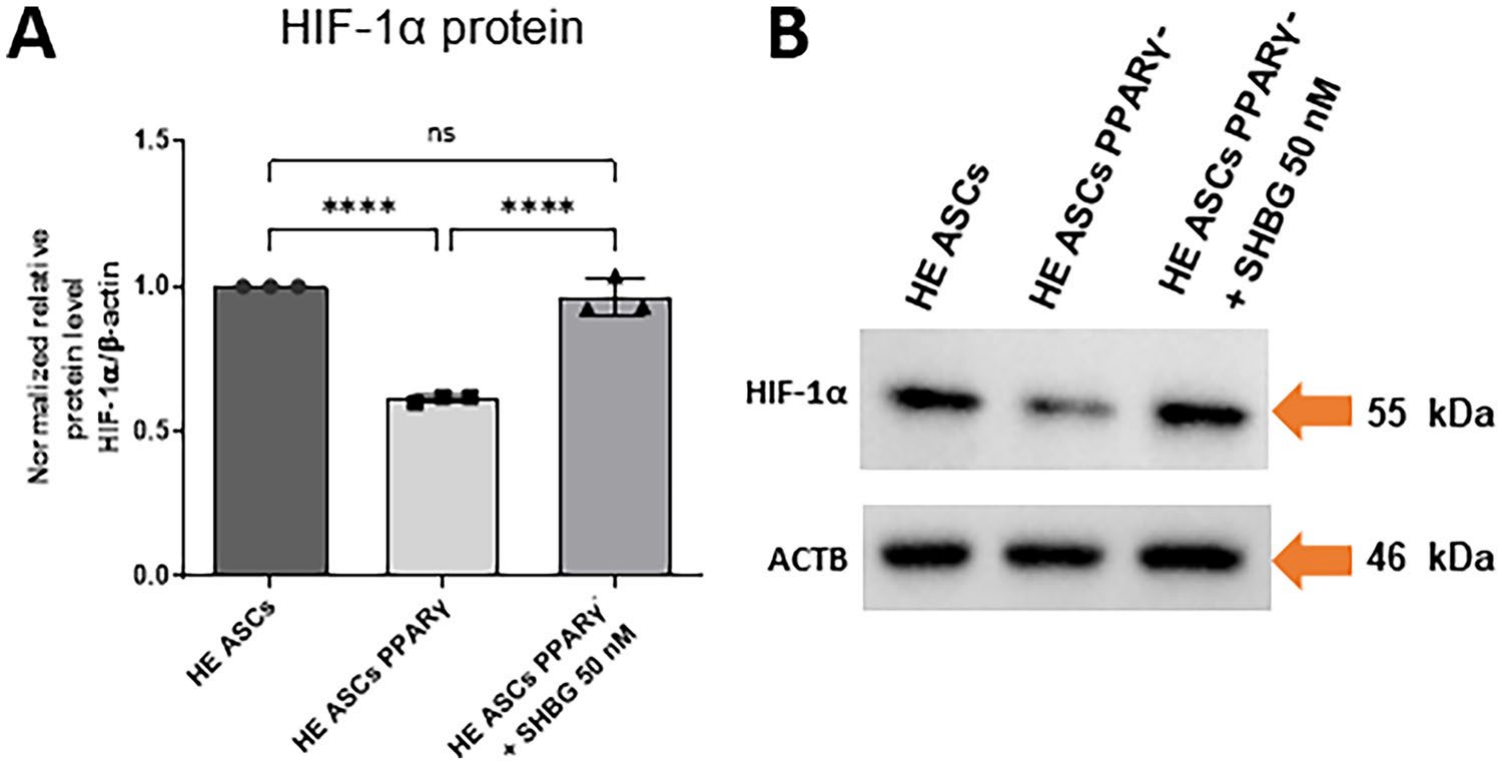
Changes in HIF1α protein levels in healthy adipose-derived stromal cells (ASCs), PPARγ-silenced ASCs, and PPARγ-silenced ASCs after incubation with 50 nM SHBG. **A** Proteins level calculated using Image Lab software after normalization to β-actin as an internal protein control. **B** Representative Western blots; results are expressed as mean ± SD. Statistical significance is marked with asterisks (*): ****p < 0.0001

The level of the HIF-1α protein (detected as 55 kDa band, suggesting the presence of degraded HIF 1α [22]), which is sensitive to the concentration of oxygen in the cells, was statistically significantly lower in cells with PPARγ gene silenced compared to native cells. Incubation of PPARγ-silenced cells with SHBG resulted in a significant increase in HIF-1α protein expression to a level similar to that of the native group (Fig. 8A).

## Discussion

Peroxisome proliferator-activated receptor-gamma (PPARγ) is a pleiotropic ligand-activated transcription factor belonging to the nuclear hormone receptor gene superfamily, which is implicated in the modulation of lipid and glucose metabolism, apoptosis and inflammatory responses when stimulated with small lipophilic ligands [23, 24]. In adipose tissue, PPARγ controls the mesenchymal stromal cells (MSCs) commitment to particular lineages and has been therefore referred as a master regulator of adipogenesis, since its depletion has been reported to dramatically hamper adipogenic potential of MSCs [25]. Recently, sex hormone–binding globulin (SHBG) liver protein has emerged as a determinant metabolic factor, intervening in the regulation of de novo lipogenesis and lipid metabolism in the liver, through a G-protein-coupled second messenger system that activates the cAMP/PKA axis [26, 27]. Although SHBG in its unbound form has been proposed to exert a wider range of biological functions, the exact underlying mechanisms remain still poorly studied. Hence, the crosstalk between SHBG and PPARγ factors in regulating ASCs metabolic functions has been investigated in a model of PPARγ-knockdown ASCs. The obtained data demonstrated that in the absence of PPARγ, ASCs are characterized by a critical mitochondrial failure, which is subsequently alleviated following exogenous SHBG application, showcasing the potential role of SHBG in compensating PPARγ-dependant signalling pathways.

Under abnormal metabolic states, MSCs especially those deriving from adipose tissue isolated from various species such as rats, humans, and horses have been reported to exhibit impaired proliferation, increased apoptosis, and upregulated expression of pro-apoptotic markers including TP53, BAX, and p21 in relation to the characteristic lipid metabolism imbalance, oxidative stress, and persistent metaflammation [28, 29]. Previous lines of evidence have highlighted the important role of PPARs including PPRAγ in the regulation of cell proliferation and survival under physiological and stress conditions [30–32]. In this study, we found that PPARγ silencing triggered a significant reduced cell viability and increased early and late apoptosis in equine ASCs. Similar outcomes have been obtained by Karnik and collaborators [33], who demonstrated that specific PPARγ deletion in murine hair follicle stem cells results in increased expression of apoptosis-related genes CASP3 and DUSP11 and consequent reduced cell viability. PPARγ silencing in renal tubular cells has also been reported to promote apoptosis through overexpression of BAX, activation of caspase 3, and increased cytochrome C leakage [34]. Accordingly, lower PPARγ expression and depleted PPARγ/Pi3K/Akt pathway have been correlated with excessive BAX protein activation, cytochrome C release, and overproduction of nitric oxide (NO) contributing to pancreatic cell apoptosis [35]. The mechanisms by which PPARγ loss leads to aberrant apoptosis and reduced cell survival have been associated to altered lipid metabolism and accumulation of stress and pro-inflammatory molecules such as reactive lipids and ROS deriving from the 5-LO and COX2 pathways, that specifically activate a lipid-mediated programmed cell death [33]. Interestingly, treatment of PPARγ-knockdown ASCs with exogenous SHBG neither ameliorated cell viability nor lowered the percentage of apoptotic cells. These observed data are in agreement with previous findings that evidenced the implication of SHBG in various cancer cell proliferation inhibition. Lee and Hong [36] demonstrated that SHBG suppresses human hepatocarcinoma cancer cell proliferation by targeting JNK/p38/ERK/AP-1 signals. Similarly, SHBG protein has been shown to strongly impede MCF-7 breast cancer cell survival and proliferation by inhibiting the phosphorylation of Erk-1/-2 [37]. By contrast, another recent report has indicated that SHBG maybe a critical survival mediator of non-cancerous cells. Indeed, Li et al. [38] demonstrated that SHBG loss results in overexpression of caspase-3, depleted pro-survival Bcl-2 protein and increased apoptosis in human trophoblast cells. These conflicting results thus hint that SHBG may exert differential effects that depend on the cell type as well as its biological and metabolic status. Hence, our obtained data suggest that SHBG may not influence cell survival of PPARγ-deficient equine ASCs, as neither amelioration nor exacerbation of cellular apoptosis following PPARγ silencing have been noted.

The gradual loss of longevity in MSCs triggers various cellular dysfunctions associated with aging including the accumulation of damaged DNA, epigenetic alterations, reactive oxygen species (ROS), mitochondrial dysfunctions as well as a profound loss of proteostasis, that collectively lead to cellular senescence and proliferation arrest [39]. PPARγ implication in regulating aging-related metabolic dysfunctions has been previously reported. PPARγ deficiency triggers an exacerbation of age-associated obesity and metabolic failure in the subcutaneous adipose tissue of aged mice [40]. Likewise, lower expression levels of PPARγ induce declined lifespan in lipodystrophic PPARγ 1/2-hypomorphic and deficient mice [41]. Besides in the present study, we observed that PPARγ-silenced ASCs displayed increased SA-β galactosidase activity as a prominent hallmark of cellular senescence establishment, evoking the critical protective effect of PPARγ toward premature cell aging. In concordance with our observations, Briganti et al. [42] similarly reported that PPARγ up-modulation prevents the biological modifications associated to the senescence-like phenotype in a stress-induced premature senescence (SIPS) model in human dermal fibroblasts exposed to 8-methoxypsoralen plus + ultraviolet-A-irradiation (PUVA), mainly by promoting mitochondrial functions and endogenous antioxidant defences, which is consistent with our obtained results that showed a critical downregulation of antioxidant markers CAT and NRF2 following PPARγ downregulation. Therewith, Pompili and colleagues [43], evidenced the correlation between PPARγ activation and mitigation of senescence markers including β-gal, laminin b1, and SASP members in colonic mucosa biopsies obtained from patients with inflammatory bowel disease, and in a mouse model of colitis induced by dextran-sodium-sulphate. Remarkably, the application of exogenous SHBG to PPARγ-silenced ASCs resulted in a significant decreased in the number of SA-β gal-positive cells and specifically increased the expression of CAT and SOD2 antioxidant enzymes, evidencing a possible reversed senescent phenotype. To the best of our knowledge, this is the first report on the potential anti-aging property of SHBG in the cellular senescence model. However, SHBG has already been reported to modulate other molecular responses that are associated to cellular senescence. Yamazaki and collaborators [44] demonstrated that SHBG attenuates pro-inflammatory responses in LPS-stimulated murine adipocyte progenitor cells and peritoneal macrophages, by suppressing the expression and release of MCP-1, TNFα, and IL-6 and inhibiting the phosphorylation of JNK1/2 and ERK1/2. Considering that senescent cells can drive the priming of the senescence-associated secretory phenotype (SASP) consisting of pro-inflammatory cytokines such as IL-1α/β, IL-6, and MCP-1 chemokine, it can be speculated that SHBG may protect against senescence through its anti-inflammatory potential [45]. Moreover, the same study pointed out the ability of SHBG to lower the overaccumulation of reactive lipids in preadipocytes and to regulate the lipid metabolism-related markers, which could account for its anti-aging effects as far as excess lipids storage, upregulated lipid biosynthesis, and de-regulated lipid breakdown have been proposed as critical players on cellular senescence [46]. In line with this, SHBG-treated ASCs exhibited also an increased BAX/Bcl-2 ratio compared to untreated native and silenced cells. This finding is of interest for understanding the mechanisms by which SHBG could modulate senescence. As a matter of fact, a number of former investigations established a particular apoptosis-resistant phenotype of senescent cells. Yosef et al. [47] identified the BCL pro-survival protein family as a central contributor of senescence-associated apoptosis resistance and showed that suppression of Bcl-2 expression using its corresponding siRNA-induced apoptosis of human senescent fibroblasts. In like manner, Kumar and colleagues [48] showed that pharmacological suppression of the anti-apoptotic protein Bcl-2 accumulation, promoted apoptosis-mediated cell death in a model of H_2_O_2_-induced premature senescence in 3T3-L1 preadipocytes, which overall reduced the senescence-associated β-galactosidase activity and the progression of senescence-associated secretory phenotype. Taken together, it can be speculated that SHBG may restore PPARγ protective effect toward premature cellular aging by attenuating apoptosis resistance and potentially improving endogenous antioxidant defences.

The MSCs therapeutic competence is governed by the integrity of their organelles, as any stress-induced damage to organelles triggers profound MSCs failure. Mitochondria have been extensively studied for their fundamental role in maintaining the homeostasis and regulating the fate of the MSCs. Hence, mitochondria have been implicated in the regulation of stem cell self-renewal, multi-directional differentiation, survival, aging, and immunomodulatory potential [49]. Consecutively, PPARγ is reported to actively participate in mitochondrial health and function regulation. The nuclear receptor mainly coordinates nuclear and mitochondrial gene expression involved in mitochondrial biogenesis, morphogenesis, and dynamics, regulates mitochondria-mediated fatty acids oxidation, and enhances energy metabolism [11]. In the present study, PPARγ silencing resulted in a general collapse in mitochondrial integrity of equine ASCs. Thence, a loss in mitochondrial transmembrane potential and expression of key mediators involved in mitochondrial dynamics, mitophagy, biogenesis, and energy metabolism regulation has been observed in cells lacking PPARγ expression. These findings corroborate with a previous functional study conducted by Sharma and colleagues [50], who demonstrated that PPAR silencing in endothelial cells leads to profound mitochondrial dysfunction by accentuating transmembrane depolarization and ROS production, lowering the cellular ATP levels and hampering the eNOS/Hsp90 axis, critical for energy metabolism regulation. Likewise, PPARγ has also been shown to rescue damaged stressed mitochondria by reversing the low transmembrane potential and depleted oxidative phosphorylation [42]. Thus, PPARγ has been proposed as a critical mitochondria rejuvenating factor that promotes structural and functional recovery under excessive oxidant stress [51]. Here we have found that treatment of PPARγ-depleted ASCs with SHBG protein resulted in a visible amelioration of mitochondrial phenotype. SHBG-treated cells were thus characterized by improved transmembrane potential, restored expression of dynamics, mitophagy and energy metabolism mediators, and higher number of mitochondria exhibiting twisted tubular shape. The beneficial effect of SHBG protein onto mitochondrial functions has not been demonstrated previously; however, recent lines of evidence have suggested potential links between SHBG and mitochondrial function. Mitochondrial dysfunction associated to liver metabolic imbalance and disrupted lipid homeostasis has been associated to sex hormones imbalance and low SHBG levels. Accumulation of reactive fatty acids leads to mitochondria β-oxidation imbalance that subsequently contributes to liver inflammatory injury and increased expression of proinflammatory cytokines interleukin-6 (IL-6), tumor necrosis factor-α (TNF-α), MCP-1, and IL-1β, that further exacerbate SHBG depletion [52]. Noteworthy, estrogen (E2) has been found to modulate various aspects of mitochondrial activity including ATP production, transmembrane potential, biogenesis, and dynamics. Hence, the activity of E2 strictly depends on its bioavailability, which is essentially regulated by SHGB as a primary sex hormones carrier and regulator [53]. Therefore, it can be assumed that SHBG can also modulate mitochondrial biology and could potentially complete the molecular mechanisms by which sex hormones impact cellular functions. Other investigations reported on the great potential of PPARγ agonists in improving mitochondrial functions. Zolezzi et al. [54] found that ciglitazone protects neuronal mitochondria from excessive oxidative stress and modulates mitochondrial fusion-fission machinery by regulating the expression of DRP1 and FIS-1. Rosiglitazone and Pioglitazone, two other PPARγ ligands have been demonstrated to increase mitochondrial energy metabolism and mitochondrial biogenesis, through the promotion of PGC1α, NRF1, TFAM, cytochrome c oxidase subunit CO-I, and CO-IV expression [55].

Peroxisome proliferator-activated receptor-gamma coactivator-1 alpha (PGC1α) is one of the principal factors involved in the regulation of mitochondrial functions, thought its action on mitochondrial transcriptional machinery, biogenesis, and metabolism [56]. Moreover, PGC1α has been shown to restrain apoptosis by lowering ROS generation and enhancing mitochondrial homeostasis in mice [57]. PGC1α expression is coordinated by PPARγ activation, which in combination initiate the transcription of genes as well as the activity of several critical transcription factors required for proper mitochondrial function [58]. Here, the loss in PCG1α transcript following PPARγ depletion has been significantly restored by SHBG protein. Similarly to our observations, the positive effect of SHBG toward PCG1 α has already been reported in a previous study of Saez-Lopez and collaborators [59], who demonstrated that human SHBG transgenic mice, expressing high SHBG levels were characterized by increased expression levels of PCG1α at both mRNA and protein levels, underlying the ability of SHBG to restore PPARγ effects by activating the transcription of its genes targets including mitochondrial PCG1α.

Another interesting finding lies in the observed loss in hypoxia-inducible factor-1α (HIF-1α) expression following PPARγ knockdown. HIF-1α is an important transcription factor which mediates adaptive responses under cellular stress including hypoxia and oxidative imbalance [60]. In MSCs, HIF-1α has been reported as a crucial factor that maintains their metabolic flexibility and immunomodulatory properties, as overexpression of HIF-1α has been correlated with higher immunosuppressive potential and increased secretion of anti-inflammatory mediators including IDO, COX2, PD-L1, and EVs [61]. Although various investigations have highlighted a negative feedback in which PPARγ abrogates HIF-1α activation in various models of cardiomyocytes and pulmonary vascular cells [62–64], here we inversely found that PPARγ loss significantly attenuates the expression of HIF-1α protein in equine ASCs. This result stands in a good agreement with Nakashima et al. [65], who indicated that HIF-1α-PPARγ axis is necessary for maintaining human induced pluripotent stem cells survival and proliferation capacity, suggesting that in MSCs, HIF-1α, and PPARγ coordinate important pathways involved in stem cells functions. Other reports suggest that MSCs exhibit high HIF-1α -mediated glycolytic rates under normoxia that supports differentiation commitments. Here we demonstrated that SHBG treatment restored basal levels of HIF-1α in PPARγ-silenced ASCs. While there is currently no direct and well-established link between SHBG and HIF-1α, some evidences suggest that lower levels of SHBG converge with decreased HIF-1α levels and activated hypoxia is accompanied with increased expression of SHBG at both mRNA and protein levels [66, 67], which may shadow a potential crosstalk between SHBG activity and HIF-1α availability, that would need to be further verified.

## Conclusion

Overall, the present study evidenced the critical role of PPARγ in modulating ASCs functions, survival, longevity, and mitochondrial metabolism. The transfection of equine ASCs with PPARγ siRNA resulted in a reduced cell viability, the development of a premature senescence phenotype, and a profound mitochondrial failure. Furthermore, the outcomes of this investigation demonstrated that SHBG may exert PPARγ mimicking effects, as treatment of ASCs silenced cells with exogenous SHBG protein exhibited a visible reversed senescent phenotype and apoptosis resistance, improved mitochondrial transmembrane potential and restored expression of master regulators of mitochondrial dynamics and metabolism. Noteworthy, SHBG treatment substantially improved the expression of PCG1a, which is naturally activated by PPARγ, and normalized the protein expression of HIF-1α, an important factor that supports proper MSCs functions. Therefore, the presented findings suggest that SHBG may serve as a potential PPARγ-like analog that mimics its biological effects, and thus could be considered as a valuable therapeutic agent for the modulation of mitochondrial activity and the amelioration of MSCs functions. However, further in-depth studies are necessary to elucidate the exact mechanisms underlying the observed SHBG effects and whether they could be attributed to a PPARγ surrogating pathway.

## Data Availability

All datasets generated and/or analyzed during the current study are presented in the article, the accompanying Source Data or Supplementary Information files, or are available from the corresponding author upon reasonable request.
